# Information multiplexing from optical holography to multi-channel metaholography

**DOI:** 10.1515/nanoph-2023-0605

**Published:** 2023-11-27

**Authors:** Andrés Márquez, Chi Li, Augusto Beléndez, Stefan A. Maier, Haoran Ren

**Affiliations:** I.U. Física Aplicada a las Ciencias y las Tecnologías, Universidad de Alicante, P.O. Box 99, 03080 Alicante, Spain; Dpto. de Física, Ing. de Sistemas y Teoría de la Señal, Universidad de Alicante, P.O. Box 99, 03080 Alicante, Spain; School of Physics and Astronomy, Faculty of Science, Monash University, Melbourne, Victoria 3800, Australia; Department of Physics, Imperial College London, London, SW7 2AZ, UK

**Keywords:** analog holography, digital holography, holographic multiplexing, metasurface holography, orbital angular momentum of light

## Abstract

Holography offers a vital platform for optical information storage and processing, which has a profound impact on many photonic applications, including 3D displays, LiDAR, optical encryption, and artificial intelligence. In this review, we provide a comprehensive overview of optical holography, moving from volume holography based on optically thick holograms to digital holography using ultrathin metasurface holograms in nanophotonics. We review the use of volume holograms for holographic multiplexing through the linear momentum selectivity and other approaches and highlight the emerging use of digital holograms that can be implemented by ultrathin metasurfaces. We will summarize the fabrication of different holographic recording media and digital holograms based on recent advances in flat meta-optics and nanotechnology. We highlight the rapidly developing field of metasurface holography, presenting the use of multi-functional metasurfaces for multiplexing holography in the use of polarization, wavelength, and incident angle of light. In the scope of holographic applications, we will focus on high bandwidth metasurface holograms that offer the strong sensitivity to the orbital angular momentum of light. At the end, we will provide a short summary of this review article and our perspectives on the future development of the vivid holography field.

## 1 Introduction

The year 2021 saw the 50th anniversary of the Nobel Prize for Dennis Gabor, awarded “for his invention and development of the holographic method” [1]. Many advances have taken place since he published his seminal papers in 1948 and 1949 [2, 3], making holography a useful technology with important applications in optics and photonics, as described in classical books but also in recent reviews [4–8]. Most importantly, for the goal of this review, holography has shaped the way we think about information storage and display [9, 10] and information processing and transmission in many fields of science and technology [6, 11]. It has led to powerful physical insights into the light–matter interactions harnessing not only the amplitude, but also the phase and/or polarization degrees of freedom of an optical beam. It introduces a new way to record desired optical information onto the wavefront of light, which can be registered in a material platform [12], called holograms, which can then be used to modulate an incident light beam to retrieve and/or process the information.

New milestones [13] were added in 1962 within the framework of analog optical holography by the work on off-axis holography by Leith and Upatnieks [14] and reflection holograms by Yuri Denisyuk [15], the latter with similarities to Lippman’s color photography [16], using the first lasers and light sensitive materials for wavefront storage and modulation. Computer-generated holography (CGH) came into play with the methods such as the ones proposed by Brown and Lohmann [17] and Lesem et al. [18], generating a more versatile framework to represent information and opening the introduction of digital fabrication techniques [19]. The combination of CGH with high resolution pixelated cameras has generated fruitful advances in digital holography (DH) [20] and new holographic and imaging applications, such as digital holographic microscopy [21], information encryption [22] or comparative holography [23]. Both CGH and DH use computational approaches either to calculate the hologram that needs to be produced (CGH), or to numerically reconstruct (DH) the hologram that has been optically sampled by some sensor system, like a camera.

The digital optics fabrication approach of CGH is now encountering novel material platforms within the realm of nanophotonics, exploiting light–matter processes not possible with conventional recording materials which have greatly renewed the perspectives in holography and in information optics [24–26]. In this sense, flat optics devices made possible by resonant and broadband metasurfaces have enabled ultrathin computer-generated holograms (CGHs) [27, 28]. Apart from their benefits of ultrathin, lightweight, and high resolution, metasurface holograms can incorporate multiple degrees of freedom of light into holographic multiplexing. As such, multiple information channels can now be encoded into a single metasurface hologram, through the engineered metasurfaces sensitive to the wavelength [29–31], polarization [32–35], and incident angle [36]. Together with this, an additional degree of freedom, the orbital angular momentum (OAM) with theoretically unbounded set of orthogonal helical spatial modes, has recently been introduced to holographic multiplexing [37–41]. In terms of information management, OAM offers a useful platform to convey a higher number of information channels [42, 43]. Similarly, in analog optical holography, different multiplexing methods have been thoroughly exploited in the context of holographic data storage [9, 10, 44–46]. For instance, the angular and wavelength selectivity produced by the Bragg condition in volume holography enable holographic multiplexing stemming from the linear momentum conservation law. Other methods, such as shift multiplexing, peristrophic multiplexing, phase-coded multiplexing, polytopic multiplexing and combinations of them have also been developed [9, 47]. They have also been used in encryption of information for security applications [11, 48] and enabled the production of multi-functional elements in holographic and diffractive optical elements useful for optical interconnects [49] and for augmented-reality devices [50]. In this review article, we firstly give a comprehensive introduction to the field of optical holography, including analog holography and its multiplexing, and digital holography. We then review the metasurface concept that has been developed in the last decade as a new platform to realize ultrathin holograms and we highlight metasurface multiplexing holography, with our particular focus given to the latest OAM multiplexing holography. We believe information multiplexing using holographic metasurfaces is a rapidly evolving research field, which will continue to drive many new physics and advanced technologies.

## 2 From analog to digital holography

### 2.1 Analog holography and general concepts

Holography uniqueness stems from its capability to gather the whole complex amplitude information of a light wavefront [4, 7]. This is revolutionary, especially when compared with conventional imaging techniques, such as photography, where only the amplitude part is considered, and consequently all the phase information is lost. This capability to register and then reproduce the whole amount of complex amplitude information opened in the 1960s and 1970s very exciting possibilities in 3D image display and holographic optical elements, but also in optical information processing and holographic data storage. More importantly, for the sake of this review, directly or indirectly it is behind many of modern optics and photonics applications where spatial light modulation of the wavefront is required such as in holographic projection [51, 52], holographic optical tweezers [53], beam shaping [54], point-spread function engineering [55, 56], holographic microscopy [57], holographic metrology [58], surface wave manipulation [42, 43, 59–65], structured light [66], and multimode light shaping [67].

**Figure 1:**
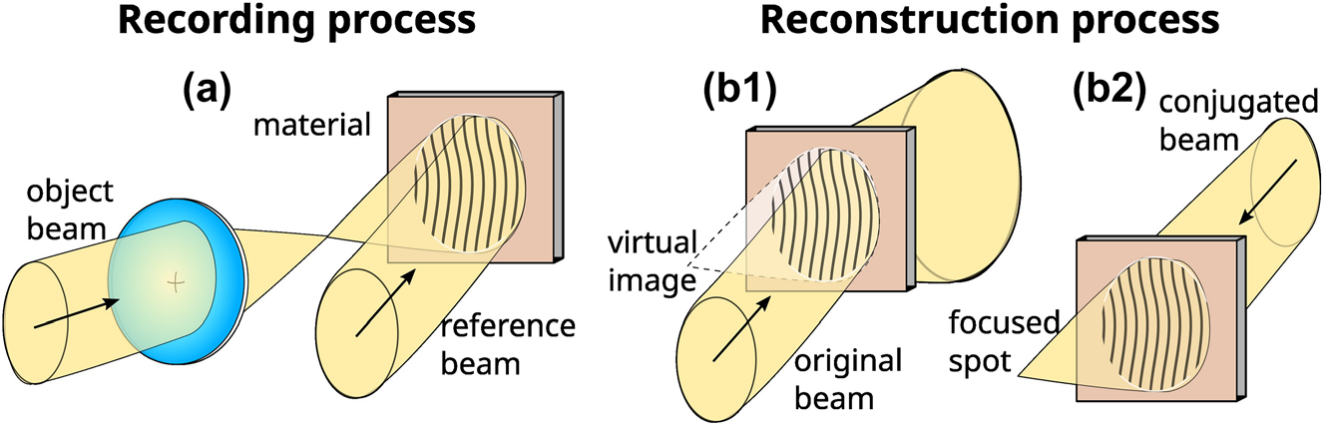
The recording and reconstruction processes of analog holography. (a) Formation of a holographic lens with a point source and an off-axis plane wave; (b1) reconstruction with the original reference wave, and (b2) with the conjugate wave.

The holographic concept is based on the combination of two separate steps, exemplified in Figure 1 for the case of a holographic lens. The first step corresponds to the recording, where the complex amplitude information of a wavefront is encoded through an interference process (Figure 1(a)). An object beam carrying the information of interest interferes with an auxiliary beam, called the reference beam. This interference pattern is translated into the modulation of the complex dielectric permittivity of an appropriate photosensitive recording material.

The second step corresponds to the reconstruction process, where an incident beam, the reconstruction beam, is diffracted/scattered by the spatially modulated material property. In general, to reconstruct the original object beam, the reconstruction beam needs to be identical to the reference beam used in the recording process, in which case a virtual image is produced (Figure 1(b1)), or to the conjugate one, where a real image is generated (Figure 1(b2)). The general process corresponds to the case of optical or analog holography, where a holographic recording material is used for the hologram. In digital holography, the interference is produced on the pixelated sensor of a camera, thus a 2D sampled interference plane is recorded [20]. In computer-generated holography [19, 68], the interference process is numerically simulated in the computer, then codified through some approach depending on the modulation properties of the registration medium. The resulting profile is afterwards implemented onto a material platform, which can be dynamic as in liquid-crystal spatial light modulators and digital mirror devices, and recently onto ultrathin metasurfaces fabricated by planar lithography and/or 3D laser nanoprinting processes.

A very important division of holograms is between thin and thick (or volume) holograms. In the latter, the thickness of the recording medium is large enough so that the reconstruction beam traverses multiple recorded interference planes. This generates the interference between the multiple diffracted waves and a coupled wave analysis is required to model the process. Furthermore, this multiple interference process is responsible for some of the most successful information multiplexing schemes. Then, in the case of a volume hologram the 3D modulation profile across the thickness of the material must be considered. When this is not necessary, we are in the range of thin holograms, also called Raman–Nath regime, where multiple diffraction orders are produced. Usually the Klein–Cook parameter *Q* is used to distinguish between these two regimes, even though this criterion can be further refined as discussed by Moharam and Young [69]. This is given by $Q=\left(2\lambda_{0}d\right)/\left(n_{0}\Lambda^{2}\right)$ , where *λ*_0_ is the wavelength in air, and *n*_0_, Λ, and *d* are respectively the average index of refraction of the medium, the fringes period and the layer thickness. In general, small values of *Q* (*Q* < 1) correspond to thin gratings, which then can be described by the Raman–Nath theory, while large values (*Q* > 1) correspond to volume gratings and its description needs more sophisticated approaches, such as the rigorous coupled-wave analysis (RCWA) by Moharam and Gaylord [70]. As a rigorous electromagnetic approach, the finite-difference time domain (FDTD) method has also been applied to analyze holographic volume gratings for the near-field distribution at optical wavelengths by Francés et al. [71, 72]. In many cases, only two of the waves are coupled significantly, the diffracted and the transmitted, as such, the simpler Kogelnik’s coupled-wave theory can be used [73, 74].

In the case of volume holograms, the multi-plane diffraction produced in the reconstruction step allows for Bragg selectivity and as a result, the reconstruction is very sensitive to the angle of incidence and/or the illumination wavelength: angle selectivity can be greatly enhanced in the case of transmission holograms and wavelength selectivity for the reflection ones [7, 44]. Volume phase holograms can reach 100 % diffraction efficiency, and if different holograms are recorded in the same volume, i.e., multiplexed, the total dynamic range of the material is shared between the different holograms. If the holograms are angularly or in wavelength detuned more than the Bragg selectivity range, then the different holograms are reconstructed independently, and the cumulative diffraction efficiency of the different holograms can even reach values greater than 100 % if the material has enough dynamic range [75]. We note that in the case of holographic data storage, where hundreds of holograms are multiplexed in the same volume, typically the diffraction efficiency and the index modulation for each hologram is very small and the Born approximation can be used to model the diffraction of light in the reconstruction instead of the more involved coupled-wave approaches [9].

### 2.2 Holographic recording media

In the recording step, the constructive and destructive interference planes between the interference wavefronts generate a spatial modulation of the index of refraction and/or the absorption coefficient across the 3D volume of a recording material. The material can also undergo superficial thickness modulation. In the cases of index of refraction and/or surface relief modulations the result is a phase hologram, generally the interesting ones since they are much more efficient than amplitude (absorption) holograms. An important distinction is also between transmission and reflection holograms, where in the former the interfering beams come from the same side of the material (as in Figure 1(a)), and in the latter, from opposite sides.

In general, the photosensitive materials used in analog holography must be able to register the very high spatial frequencies produced by the interfering beams in the recording process, typically larger than 2000 and 4000 lines/mm, respectively, for transmission and for reflection holograms. These include silver halide films, dichromated gelatin, photorefractive crystals, photopolymers, photoresist, thermoplastics, embossed holograms, and photosensitized glass [7, 12]. Some of the materials have been traditionally used in display holography (silver halide films, dichromated gelatin), then other mostly found in mass replication for security applications (photoresist, embossed holograms), and in holographic interferometry (thermoplastics). In most of the materials, a phase hologram is obtained, typically based on index of refraction modulation. Exceptions are photoresist and embossed holograms, where a surface relief modulation is produced, and silver halide films, in which a bleaching step is necessary to generate an index of refraction modulation, thus a phase hologram, from what in principle is an absorption modulation hologram [4, 7]. Nowadays, there is a very active interest in nanocomposite materials, in which nanoparticles or other kind of additives or organic molecules, such as liquid crystals (LC), are added to enhance specific properties of the material [12, 76]. In all the cases, to characterize the holographic recording materials, plane wave interference is used, and the diffraction efficiency of the resulting gratings serves to determine the values for the refractive index modulation, Δ*n*, for a phase grating, or the absorption modulation, Δ*α* for an amplitude grating.

Each holographic application generally has different requirements on the material properties. For holographic data storage, where the most sophisticated multiplexing schemes are used, as shown in the next Section, the material is required to store hundreds or even thousands of data pages in the same region of the material [77, 78]. For this, there is a need for a large dynamic range of the material response. Then, to diminish crosstalk between different channels/data pages, thick material layers are needed, with good transmission, layer homogeneity, and longtime stability. Shrinkage should be minimal to avoid degradation in the reconstruction process [79]. The materials used are basically two: photorefractive crystals for erasable media and photopolymers.

Among the different materials, photopolymers are probably the most appealing ones since they can be highly customized to different applications [80, 81]. Different photopolymer compositions have been developed, typically including one or more polymerizable monomers, a sensitizer or photo-initiator, and a binder which serves as the matrix where the reaction takes place, and additional components (plasticizers, inhibitors, and stabilizers) can be added. Acrylamide monomers in polyvinyl alcohol (PVA) binder have been extensively applied in holographic data storage multiplexing [82]. Another interesting material is glass-like phenanthrenequinone doped PMMA photopolymer (PQ/PMMA), which shows high stability and negligible shrinkage with a high storage density of 40.5 GB/cm^3^ [83]. Improvements on different holographic properties are possible by doping with different nanoparticles such as SiO_2_ or ZrO_2_, which produce an increased thermal and shrinkage stability in (meth)acrylate photopolymers [84], or gold, which increases the photoinduced birefringence in PQ/PMMA for polarization-angle multiplexing [85]. In the last decade, a commercially available photopolymer, the Bayfol^®^ HX film has demonstrated excellent properties in terms of high diffraction efficiency, polychromatic sensitivity, good durability, and flexibility for on-demand applications. They are based upon an orthogonal two-chemistry formulation, where the first chemistry defines the matrix network in which the second chemistry, with the components responsible for the hologram formation during exposure, is embedded [86].

There are also rewritable and tunable photopolymers, which include photorefractive polymers [87] and holographic polymer-dispersed liquid crystals (H-PDLC) [76], which can be useful for a variety of applications such as dynamic displays and holographic data storage. Holographic grating formation in organic photorefractive materials results from three processes: photogeneration of charge carriers, charge transport, and formation of the locally varying refractive index modulation. In the case of H-PDLCs, a hologram is recorded in the conventional manner in a solution of sensitized monomer and LC materials. An index modulation results from the exposure induced phase separation between (exposed) polymer rich and LC rich (unexposed) regions. In the latter, the LC is grouped in subwavelength droplets, in which the LC director can be reoriented by applying an electric field, thus the modulation of refraction index can be tuned. Now, in the next Section, we focus our attention on the different multiplexing schemes used in analog holography, which in many cases find their counterparts in digital holography.

### 2.3 Holographic multiplexing in analog holography

In analog holography, holographic data storage (HDS) is the research area which best exemplifies the development of information multiplexing approaches. Many efforts have been dedicated to HDS since the inception of holography, with the promise of establishing a unique approach to data storage with inherent parallelism when compared with bitwise approaches in data storage [45, 88, 89], providing the largest storage densities, highest data transfers, long archival storage, and energetically very efficient. In HDS, digital data to be stored is encoded onto the amplitude and/or phase distribution of a page-wise light wavefront, leading to parallelism, using a spatial light modulator (SLM) [90, 91], which provides more than one million pixels, thus more than 1 Mbit per stored page. Recording of multiple volume holograms in the same material region, thus leading to increased storage capacity and density, is possible through a wide variety of multiplexing techniques. In his seminal paper [92], van Heerden pointed out that the theoretical optical storage density in a volume *V* of recording material is proportional to *V*/*λ* ^3^, where *λ* is the wavelength of light. Therefore, in a volume of 1 cm^3^, an incident wavelength of 1 μm can store up to a terabit of information in theory. In fact, page oriented holographic storage systems have demonstrated the highest storage density of any removable media (>700 Gb/in^2^) and are theoretically capable of achieving up to 40 Tb/in^2^ [78].

**Figure 2:**
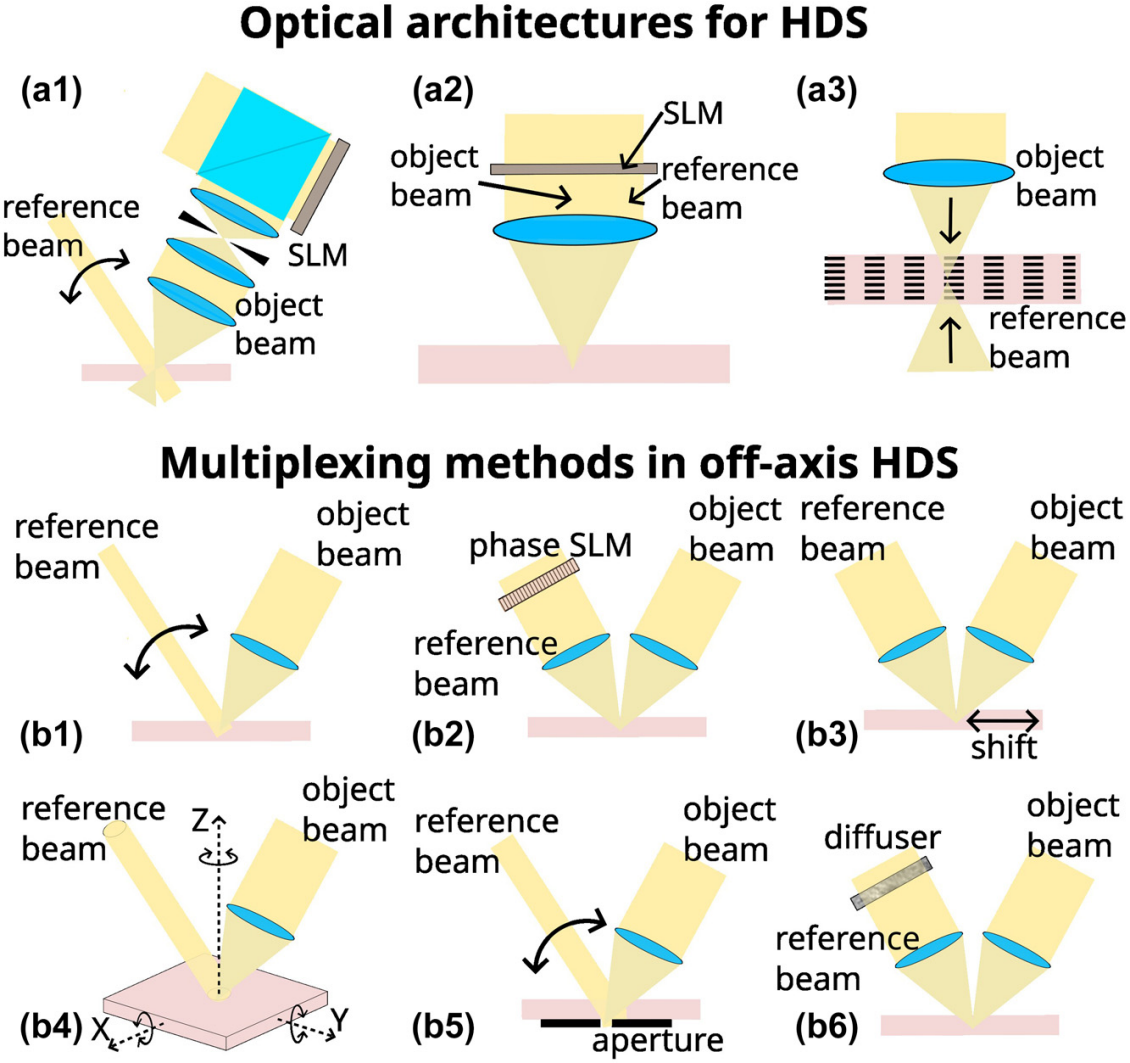
Optical architectures and multiplexing methods in holographic data storage (HDS). (a) Representative optical architectures for HDS systems. SLM: spatial light modulator. (a1) 2-axis geometry, (a2) collinear, and (a3) microholographic storage. (b) Scheme for multiplexing methods: (b1) angular, (b2) phase-coded, (b3) shift multiplexing with a spherical reference beam, (b4) peristrophic, indicating 3 different possible rotations, (b5) polytopic, and (b6) correlation-speckle.

In addition to the multiplexing scheme, there are different optical architectures that have been proposed along the years, which can be generally divided in three groups as proposed by Ruan [45], shown in Figure 2(a). The optical architecture for 2-axis HDS (Figure 2(a1)) generally has two optical paths for an object beam and a reference beam with an off-axis optical configuration, which is the general scheme presented in the most of previous sections. For the collinear HDS [93] (Figure 2(a2)), the information beam and a reference beam are produced simultaneously by the same SLM, with the information pattern in the center and reference pattern as a ring around it and interfere with each other in the holographic media through a single objective lens. In the reconstructing process, only the reference pattern, on the outer ring, is used for creating a reference beam. For micro-holographic storage [94] (Figure 2(a3)), two counter-propagating object and reference beams are configured to record bitwise information similar to the DVD system. Microscopic reflection holograms correspond to the single bits, and they are afterwards recovered when illuminating through the object beam path. High storage densities can be achieved by combining multiplexing methods and multilayer storage at each bit location.

In the case of the 2-axis and collinear geometries, in general, a Fourier transform hologram is recorded by placing the SLM aperture at the front focal plane of a lens and the holographic recording material at the back focal plane. Under this scheme, since it records the spatial frequency content of the data page, non-essential spatial frequencies can be discarded with a spatial filter, so-called Nyquist aperture, minimizing the recording area on the material. It is also more robust to misalignments since the Fourier transform amplitude is shift-invariant, and also more robust to small imperfections or dust on the SLM or material plane since this only produces slight decrease of the signal-to-noise ratio.

Regarding the multiplexing methods, they can be divided into three underlying physical principles, as described by Curtis et al. (Chapter 2 in Ref. [47]): Bragg-based, momentum-based, and correlation-based methods. These methods are listed in Table 1 with some of their more relevant characteristics, and some are shown in the diagrams in Figure 2(b), based on the 2-axis optical architecture. Multiplane-scattering processes, responsible for volume holograms (explained in Section 2.1), are behind the Bragg-based methods, in which only one object/data hologram is constructively generated for each specific reconstruction beam. In its basic format the reference/reconstruction beam is a plane wave, as it is the case in angle, wavelength and peristrophic multiplexing. In the case of phase-code and shift multiplexing the reference/reconstruction beams are orthogonal phase-patterns and a spherical wavefront respectively. The directionality of diffraction, i.e., k-vector beam direction, is responsible for momentum-based methods, in which Bragg selectivity is very low. Therefore, various holograms are simultaneously reconstructed with similar intensities but only one on the appropriate direction of the camera sensor. Then, a limiting aperture is used at an appropriate plane to filter out unwanted reconstructions. The correlation methods depend on the specific spatial and polarization structure of the reference fields employed, which is typically some pseudorandom pattern. This makes, that for the reconstruction, a slightly different pattern from the one used in the recording, does not produce a constructive scattering. This can be used to encrypt information in the holographic memory, but also to produce an even larger selectivity than the one given by Bragg-scattering in volume holography. We note that in the case of peristrophic multiplexing, depending on the direction of the media rotation, this produces Bragg-selectivity or simply affects the k-vector direction. In Figure 2(b4), where the interfering beams are along the *YZ* plane, the turn around the *X*-axis generates recording with Bragg-selectivity, but the turns around the *Y*- and the *Z*-axis do not. Furthermore, various multiplexing methods can be combined to maximize the storage capacity of the material [105], such as angle and peristrophic or shift and aperture [47].

**Table 1:** Summary of multiplexing methods used in holographic data storage.

Method	Main characteristics and performance	Ref.
**Bragg-based**		
Angle	>1000 holograms; in transmission; most widespread; enables combination with peristrophic and polytopic	[77, 95]
Wavelength	>1000 holograms; in reflection; requires a highly stable tunable laser and good thermal stability	[96]
Phase code	>100 holograms; in transmission; robust design with no mechanical parts but needs a second SLM and high phase modulation accuracy	[97]
Peristrophic	>100 holograms; in transmission; easy to implement	[98]
Shift	>1000 holograms; in transmission; reference is spherical wave; suitable for disc media; low selectivity in the non-Bragg direction but then it can be combined with aperture multiplexing	[99, 100]
**Momentum-based**		
Peristrophic	Material rotation orthogonal to the Bragg-selectivity plane; amplifies the capacity in angle multiplexing	[98]
Aperture	Combined with shift-multiplexing in the non-Bragg direction provides a better usage of the media capacity	[101]
Polytopic	Combined with angle-multiplexing ensures storage density to scale linearly with media thickness	[102]
**Correlation-based**		
Correlation-shift	Shift selectivity becomes larger than Bragg selectivity and not dependent on media thickness; however, noise build-up increases with the number of holograms; can be used for optical encryption	[103, 104]

We note that, as discussed in the last paragraph in Section 2.1, the cumulative diffraction efficiency of the multiplexed holograms can reach values higher than 100 %. This is true both for Bragg-based and correlation-based methods where only one hologram is constructively generated for each specific reconstruction beam. In the case of momentum-based methods the efficiency is not the most relevant parameter, but the separability between the directions of the various holograms that are simultaneously reconstructed, so that unwanted reconstructions can be filtered out.

To further increase the amount of data stored, multilevel recording methods have been proposed in which the data page displayed on a SLM is addressed with multiple levels instead of using the common digital representation with just binary amplitude values of “0” and “1”. By utilizing the gray scale amplitude values between “0” and “1”, it is possible to increase the amount of data [106]. In addition to the amplitude values, phase values can be used as information [107–109]. These encoding methods increase the coding rate of a data page, and thus increase storage capacity and data transfer rate simultaneously.

Even though multiplexing methods have been introduced in relation with HDS, some of them have also been used in other optics and photonics applications, such as in the very active fields of holographic concentrators for photovoltaic energy harvesting [110, 111] or in holographic combiners for see-through glasses in augmented reality (AR) [112]. In the case of holographic solar concentrators, different researchers have explored angular multiplexing of holographic lenses to increase the range of acceptance angles, thus avoiding expensive tracking systems [113, 114]. In AR see-through devices, holographic optical elements enable in-coupling of the virtual image generated with a micro-display onto a waveguide, where it is guided by total internal reflection, until the holographic out-coupler brings out the image onto the user eyes while permitting to see the environment in the background [112, 115]. Kim and Park [50] implemented a Maxwellian near-to-eye display with an enlarged eye-box by multiplexing multiple concave mirrors into a single holographic optical element onto a photopolymer. In the case of Odinokov et al. [116], an AR display based on a planar waveguide made in photothermo-refractive glass is demonstrated, where the monolithic integration of multiplexed volume Bragg gratings with the waveguide platform provided in/out-coupling and image transmission from a portable projector, and the multiplexing enabled enlarged field of view.

### 2.4 Digital holograms realized from diffractive optical elements

Arbitrary wavefronts can be digitally designed based on CGHs, because we are not subject anymore to the shapes of real objects and no holographic interference setup is needed for recording [7, 19]. When illuminated, diffraction by the CGHs gives rise to the desired wavefronts that can reconstruct target images at a pre-defined plane.

When designing a CGH, several parameters need to be considered. In this sense, two basic CGHs can be designed whether the propagation distance from the object to the hologram plane falls within the near- or far-field diffraction regions, which correspond respectively to the Fresnel and Fourier holograms. The sampling interval and number of points that must be encoded depend on the spatial frequency content of the object field which also depends on the type of hologram. The sampled values can be calculated with different computational methods such as the Gerchberg–Saxton and other iterative approaches [117]. In these methods, iterative diffraction propagation back and forth between the CGH plane and the image plane is calculated, and the constraint of the hologram medium is imposed in each loop until the diffracted field at the image plane converges to the desired distribution. It is important to note that diffractive optical elements can produce different functions simultaneously. Thus, a CGH can both produce the Fourier transform of a hologram and codify a lens, or produce the focusing of different illumination wavelengths at a certain plane [118]. In this sense, diffractive optics enable the multiplexing of different functions and information channels.

To implement high-resolution CGHs, a digital printer was originally proposed [119]. The availability of spatial light modulator devices has opened the possibility of dynamic CGHs which can be changed in real-time [120]. SLMs, partly introduced in previous Sections, are pixelated devices with resolution of various megapixels and pixel sixes smaller than 10 µm in modern devices, which can modify either the phase or amplitude of reflected or transmitted light. A variety of SLMs have been developed including magneto-optic, multiple quantum well, liquid crystal, and micro-electro mechanical (MEMS). The most widespread and probably most matured technologies are liquid crystal-on-silicon (LCoS) microdisplays [90] and deformable micro-mirror (DMD) devices [120].

## 3 Metasurface holography

### 3.1 Metasurface multiplexing holography

Metasurface multiplexing holography is a novel technique that enables the generation of dynamic and multi-channel holographic images using ultrathin planar optics. Metasurfaces consist of arrays of subwavelength nanostructures (meta-atoms) that can manipulate the phase, amplitude and polarization of light with high spatial resolution and flexibility. By incorporating various multiplexing methods, such as wavelength [30, 121–125], polarization [34, 126–128], OAM [38, 39, 41, 129–131], spatial position [132], nonlinear frequency conversion [133–135] and code division multiplexing [136, 137], metasurfaces can encode multiple information channels into a single hologram [24, 25]. This allows for high-capacity and versatile holographic applications in optical displays [138], data storage [46, 139, 140], encryption [37, 138, 141–143] and communication [144, 145]. Some recent advances in metasurface multiplexing holography are summarized here. Specific development dealing with OAM metasurfaces and multiplexing are dealt in the next Section.

#### 3.1.1 Orthogonal polarization multiplexing meta-holograms

**Figure 3:**
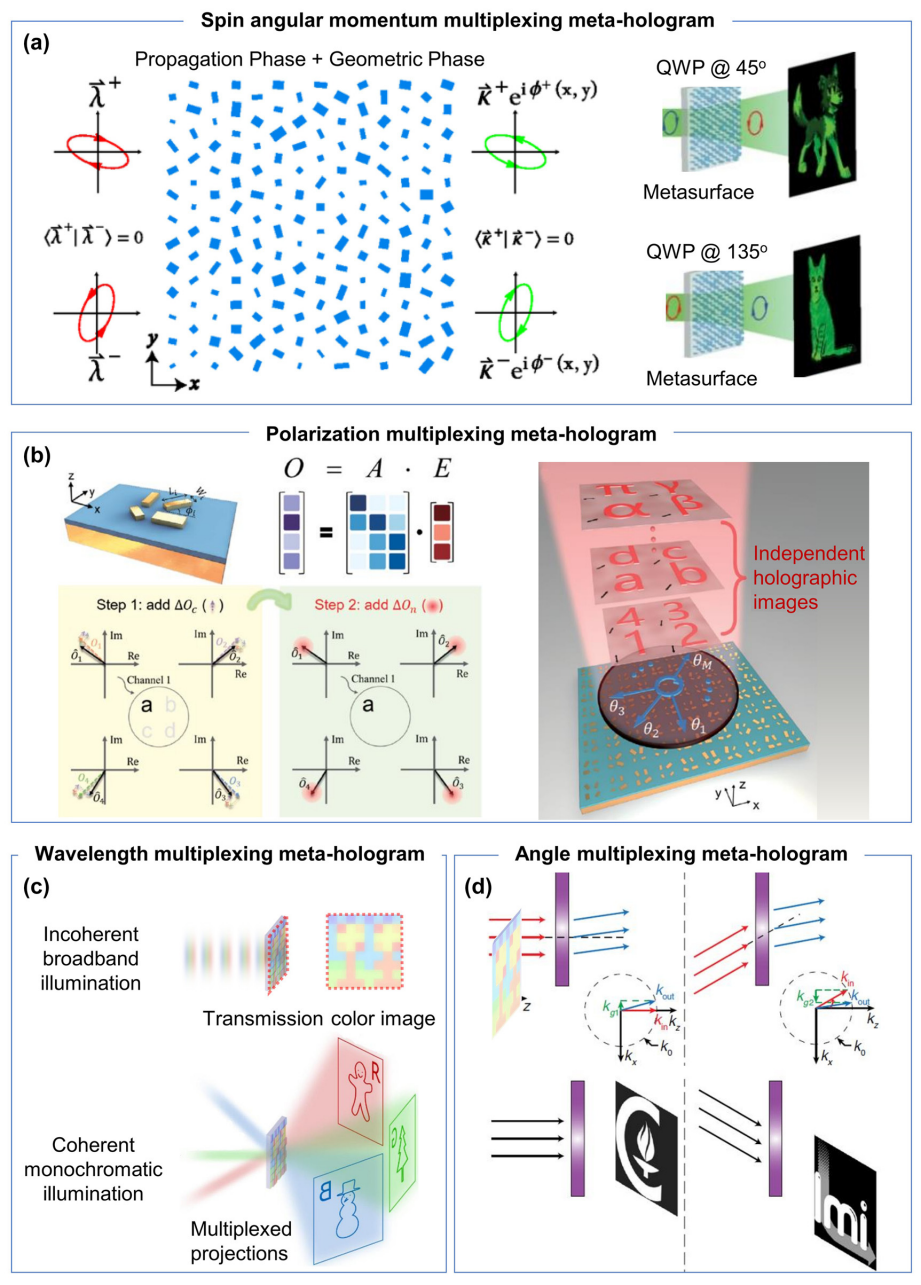
Metasurface multiplexing holography. (a) Arbitrary orthogonal polarization multiplexing meta-holograms. Left, the combined propagation (*x*–*y* orthogonal bricks) and geometric (rotated bricks) phase design. Right, experimental reconstruction of two independent image channels from an elliptical-polarization-multiplexed meta-hologram. Reproduced from Ref. [126] with permission from American Physical Society, copyright 2017. (b) High-dimension polarization-multiplexed meta-hologram. Top left, an example of unit cell and the responsive matrix. Bottom left, the design principle of polarization multiplexing through engineered correlated (step 1) and random (step 2) noise. Right, schematic of holographic display from a high-dimension polarization meta-hologram. Reproduced from Ref. [32] with permission from American Association for the Advancement of Science, copyright 2023. (c) Wavelength-multiplexed holographic color prints. The device displays as color images in white light (top) but three different holograms under RGB laser illumination (bottom). Reproduced from Ref. [30] with permission from Nature Publishing, copyright 2019. (d) Angle-multiplexed meta-hologram. Schematics illustrates those two images can be extracted out based on two different incidence angles. Reproduced from Ref. [36] with permission from American Physical Society, copyright 2017.

In 2017, Capasso group demonstrated chiral holograms with independent phase control of arbitrary orthogonal states of polarization, pushing the holographic multiplexing from circular polarizations [34] to any desired orthogonal states on the Poincaré sphere [126]. Generally, metasurfaces impart polarization dependent phase profiles through two major principles: propagation phase design for linear polarizations and geometric phase design for circular polarizations carrying the spin angular momentum. For example, geometric phase from space-variant subwavelength dielectric gratings can lead to a spin angular momentum-dependent focusing lens [146]. Considering the case of more general elliptical polarizations, neither of the two mentioned principles alone can address the polarization selectivity. As shown in Figure 3(a) left, Capasso group combined the above two principles to manipulate the phase of any two orthogonal states of polarization independently and with high efficiency. Their metasurface consists of an array of dielectric nanopillars with elliptical cross-sections that act as birefringent meta-atoms. By tuning the orientation and aspect ratio of the nanopillars, they can achieve arbitrary phase profiles for any desired orthogonal states of polarization, including the linear and circular polarization states. Experimental results of the chiral holograms are shown in the Figure 3(a) right, where under the illumination of left- and right-handed circular polarizations, a cartoon dog and a cat can be reconstructed respectively.

#### 3.1.2 High-dimensional polarization multiplexing meta-holograms

The conventional design of polarization-multiplexed metasurfaces is limited by the dimensionality of the Jones matrix, which describes the polarization transformation of light by each metasurface element [147]. The upper limit of independent channels for polarization multiplexing was theoretically limited to 3 [147]. Wang group recently reported a high-dimensional polarization multiplexing metasurface hologram that breaks this limit and achieves unprecedented polarization multiplexing capacity, which was achieved from introducing controlled noise to the each polarization image channel [32]. As shown in Figure 3(b), correlated noises (step 1) were firstly added to polarization channels, while the random noises (step 2) were further added to approach the precise solutions of Jones matrix elements which shall wipe the crosstalk of holographic images (inset alphabet image). By doing so, multiple independent polarization channels carrying independent holographic images are created. Experimentally, they demonstrated that a single metasurface can generate up to 11 independent images for different polarizations of visible light, which is the highest number reported so far. Moreover, by combining polarization multiplexing with position multiplexing, they further generated 36 distinct images from a large piece of a metasurface hologram forming a holographic keyboard pattern. It shows that noise can be beneficial rather than detrimental in optical engineering if it is properly controlled and utilized.

#### 3.1.3 Wavelength multiplexing meta-holograms

Color prints and holograms are used as two typical optical security means. Adding amplitude control to the phase holograms, one can achieve bifunctional device that displays color images under incoherent white light and different holograms under coherent laser illumination. Figure 3(c) shows a transmission-type holographic color print by Yang’s group in 2019 [30]. In this work, the encoding of phase and amplitude is realized at an individual pixel level, each pixel consists of a top-layer structural color element that modulates the amplitude of transmitted light and a middle-layer phase plate that modulates the phase of light. The structural color element is a dielectric nanopillar that transmits desired wavelength on-axis while the rest are diffracted with large angles. The wavelength selectivity stems from the nanopillar dimensions (i.e., height, diameter and pitch). The phase of transmitted light is then modified by the phase plate, following the equation $\emptyset \left(\lambda \right)=2\pi \left(n-1\right)t/\lambda$ , where *λ* is the incidence wavelength, *t* is the phase plate thickness and *n* is the medium refractive index. By arranging these pixels in an array, the device can display a colored image under white light illumination and project up to three different holograms under red, green, and blue laser illumination. This device enables good versatility and security by creating holographic color prints with various images that are readily verified but challenging to emulate. Moreover, the whole device was fabricated in a monolithic way that makes it more attractive for applications.

#### 3.1.4 Angle-multiplexed meta-holograms

Angle is another physical dimension that has been used for traditional multiplexing holography. Conventional lenses, gratings and holograms have a highly correlated angle response, meaning that the same optical transformation is applied to different incident angles with possible distortions and efficiency reduction [148]. Faraon and co-workers introduced a novel concept of angle-multiplexed metasurfaces, which can encode independent wavefronts in a single meta-hologram under different illumination angles [36]. To achieve this, the authors applied high-contrast dielectric (a-Si) U-shaped meta-atoms on a reflective substrate, whose scattering properties depend strongly on the angle of incidence. By designing the meta-atoms to have different resonant modes under different incident angles, they can control the phase and amplitude of the reflected light independently for each angle (Figure 3(d)). This enables flat optical devices that can perform different and independent optical transformations when illuminated from different directions, such as angle-multiplexed gratings and holograms.

Metasurface multiplexing holography is a promising research field that has the potential to revolutionize the field of holography and optical information processing. However, there are still some challenges and limitations that need to be addressed, such as the fabrication complexity, reduced efficiency and pronounced crosstalk through multiplexing, limited bandwidth, and the compatibility with different light sources. We next introduce another promising platform for multiplexing holography based on the use of the orbital angular momentum (OAM) modes.

### 3.2 Orbital angular momentum holography and multiplexing

High-bandwidth holograms are essential for high-capacity holographic memory devices. Owing to the use of subwavelength meta-atoms (single pixels of a metasurface), ultrathin metasurface holograms can suppress the multiple-order diffraction effect. Moreover, meta-atoms sensitive to different physical properties of light including polarization [32] helicity [34], wavelength [29], and incidence angle [36] can be designed to encode independent information channels for holographic image multiplexing. Usually, these multiplexing approaches demand for special engineering of meta-atoms, posing a challenge to increase multiplexing channels. Multimode light shaping [66, 67] raises new hope to boost the hologram bandwidth via a new degree of freedom of the orbital angular momentum (OAM) modes [37–41]. OAM holography features a huge multiplexing capacity by exploring a theoretically unbounded set of orthogonal OAM modes. Moreover, the OAM sensitivity can be realized from the computational design of the hologram without the special requirement on the meta-atoms sensitive to different OAM modes. As such, OAM multiplexing holograms can be implemented by various platforms, such as spatial light modulators, digital micromirror devices, phase-only metasurfaces, and complex-amplitude metasurfaces. Here we will go through the physical background of the OAM holography and provide a review on its recent development for high-dimensional holographic multiplexing.

#### 3.2.1 Principle of OAM holography

**Figure 4:**
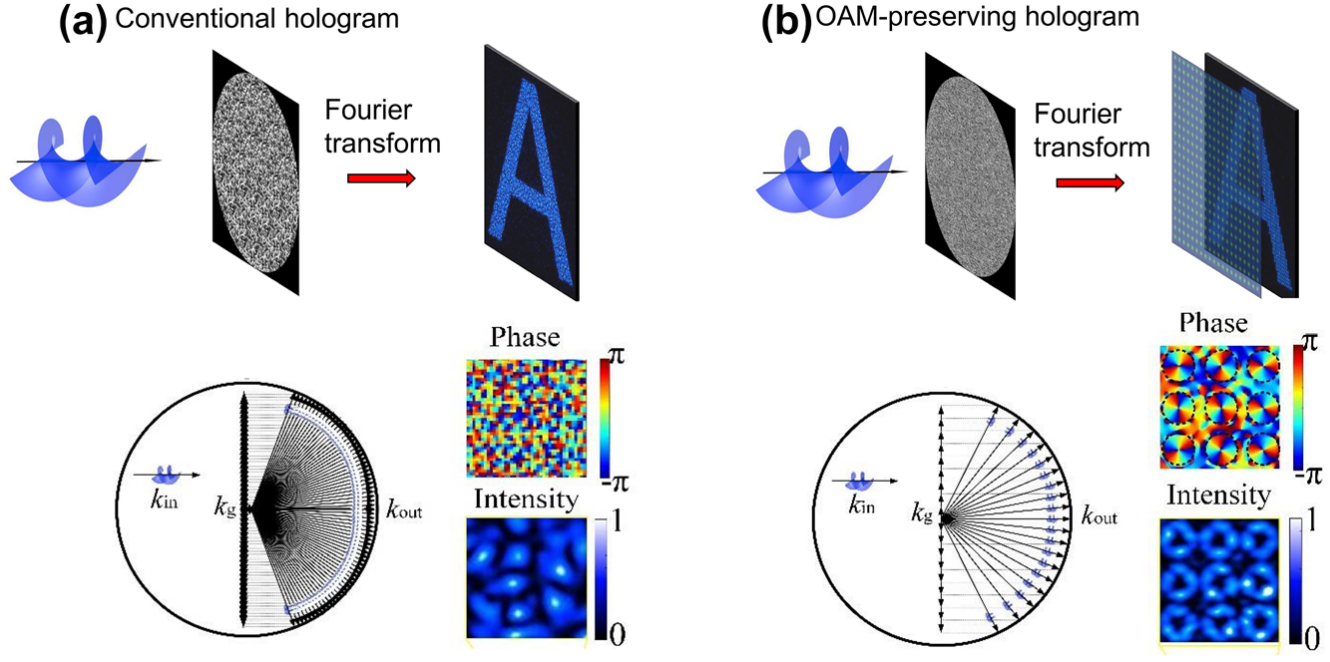
Comparison of a conventional digital hologram with an OAM-conserving hologram. (a) A conventional digital hologram with a quasi-continuous spatial frequency distribution breaks down the helical wavefront carried by an incident OAM beam. The inset (left) shows a quasi-continuous spatial-frequency content of the hologram. The insets (right) show the phase and intensity distributions of an enlarged pixel in an OAM-reconstructed holographic image, respectively. (b) An OAM-conserving hologram with a discrete spatial frequency distribution, capable of preserving the helical wavefront from an incident OAM beam. The inset (left) shows a discrete spatial frequency distribution of an OAM-conserving hologram. The insets (right) show the phase and intensity distributions of an enlarged pixel in an OAM-reconstructed holographic image, respectively. Reproduced from Ref. [38] with permission from Nature Publishing, copyright 2019.

Unlike other physical properties of light, OAM of light has very recently been explored for optical holography, mainly due to the lack of OAM selectivity in the conventional hologram design. Typically, a conventional digital hologram has a quasi-continuous spatial frequency distribution, destroying the helical wavefront of an incident OAM beam and hence losing the OAM physical property in the holographic reconstruction process (Figure 4(a)). Mathematically, the OAM-reconstructed electric field in the image plane is a convolution between the electric field of a holographic image and the Fourier transform of a helical wavefront [38]. In this case, the Fourier transform of a helical wavefront, which acts as the kernel function of the convolution, is simply copied in each pixel of the holographic image. As such, to preserve the OAM property in each pixel of a reconstructed holographic image, it is necessary to spatially sample the holographic image by using an OAM-dependent 2D Dirac comb function to avoid spatial overlap of the helical wavefront kernel, i.e., creating OAM-pixelated images. As such, the constituent spatial frequencies (*k*_g_ in the momentum space) of an OAM-conserving hologram add discrete linear spatial frequency shifts to an incident OAM beam (*k*_in_). The outgoing spatial frequencies leaving the hologram (*k*_out_) possess a helical wavefront inherited from the incident OAM beam, implying that the OAM-conserving hologram could create OAM-pixelated holographic images (Figure 4(b)).

OAM-multiplexing holography can be represented as superposition of complex-amplitude fields of different image channels encoded with distinctive OAM modes in the hologram plane: $E^{\text{mul}}=\sum_{j=1}^{M}A_{j}{\text{e}}^{{\text{i}\emptyset }_{j}}{\text{e}}^{\text{i}\varphi_{j}}$ , wherein *A*_*j*_ and ∅_*j*_ stand for the amplitude and phase information of each image channel, respectively; *φ*_*j*_ = *l*_*j*_ *φ* where *l*_*j*_ ϵ Z and *φ* represent the helical mode index and azimuthal angle, respectively, and *M* denotes the total number of multiplexing channels. Since the complex-amplitude hologram is Fourier-based, its reconstructed optical fields in momentum space can be represented as $F\left(E^{\text{mul}}\right)=\sum_{j=1}^{M}F\left(A_{j}\right)⊗F\left({\text{e}}^{{\text{i}\emptyset }_{j}}\right)⊗F\left({\text{e}}^{\text{i}\varphi_{j}}\right)$ , where F denotes the Fourier transform operator, expressing multiplexing results as the superposition of a convolution of the amplitude (*A*_*j*_), phase (∅_*j*_), and encoded OAM (*l*_*j*_) information of each image channel. It is obvious to see that the amplitude of each image channel can be individually controlled, allowing the adjustment of image frame intensity essential for high-quality holographic video displays. On the other hand, a phase-only OAM-multiplexing hologram can be described as an argument result: $P^{\text{mul}}=\arg\left[\sum_{j=1}^{M}{\text{e}}^{{\text{i}\emptyset }_{j}^{\prime }}{\text{e}}^{\text{i}\varphi_{j}}\right]$ , wherein $\emptyset_{j}^{\prime }$ denotes an iteratively retrieved phase-only hologram for each image channel. The reconstructed optical fields in momentum space can thus be represented as $F\left(P^{\text{mul}}\right)=F\left\{\arg\left[\sum_{j=1}^{M}{\text{e}}^{{\text{i}\emptyset }_{j}^{\prime }}{\text{e}}^{\text{i}l_{j}\varphi }\right]\right\}$ , indicating that the amplitude information of each image channel is lost, which degrades the reconstruction quality by increasing the multiplexing crosstalk.

**Figure 5:**
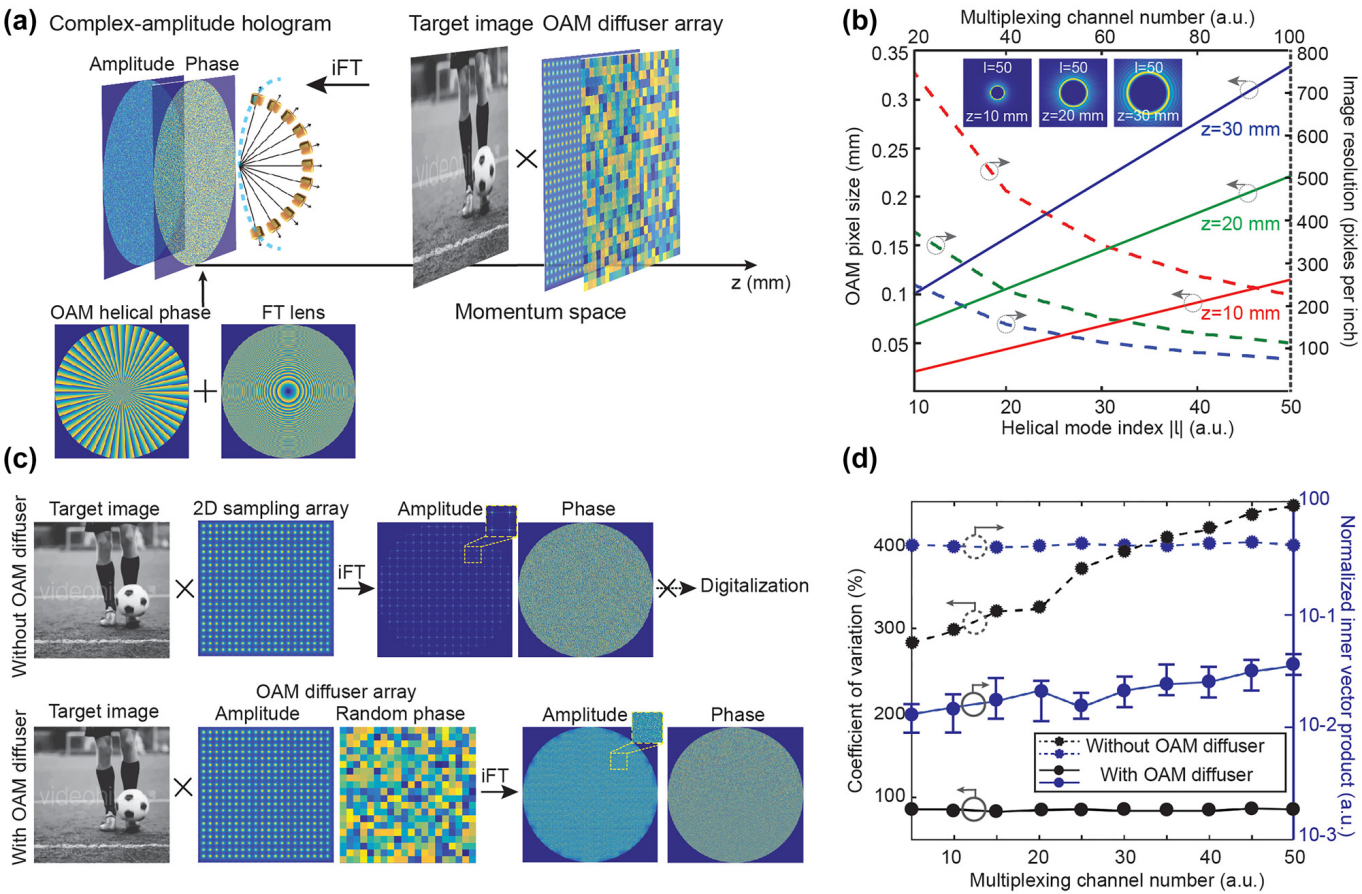
Design principle of a complex-amplitude hologram for OAM holography in momentum space. (a) Schematic illustration of OAM holography based on a complex-amplitude hologram that is sampled in its momentum space through an OAM diffuser array. OAM helical phase and a Fourier transform lens were added to the phase information of the complex-amplitude hologram, offering strong OAM selectivity at a given image plane. iFT represents inverse Fourier transform. (b) Numerical characterization of OAM pixel size and image resolution as a function of helical mode index |*l*| at different image planes, respectively. (c) The effect of an OAM diffuser array with random phase on the digitalization of a complex-amplitude Fourier hologram. (d) Numerical characterization of coefficient of variation (black lines) and normalized inner vector product (blue lines) of complex-amplitude Fourier holograms as a function of OAM-multiplexing channel number, with (solid lines) and without (dashed lines) the use of an OAM diffuser array, respectively. Reproduced from Ref. [39] with permission from Nature Publishing, copyright 2020.

The design principle of a complex-amplitude hologram for OAM holography is illustrated in Figure 5. To achieve OAM holography, an OAM diffuser array that consists of an OAM-dependent sampling array and a random phase function was developed to perform the spatial-frequency sampling in momentum space (Figure 5(a)). As such, the inverse Fourier transformation of a sampled target image gives rise to a complex-amplitude hologram with a discrete spatial-frequency content. In each holographic image channel, a specific OAM helical phase and a Fourier transform lens were added to the phase information of the complex-amplitude hologram, leading to the OAM selectivity at a given image plane. According to diffraction theory, the size of OAM pixels in the image space can be determined from both the helical mode index (*l*) at a given wavelength and the effective numerical aperture of the Fourier hologram with respect to a specific image plane. As a result, there are fundamental limits on both the maximal multiplexing channel number and the upper resolution limit of an OAM-converted holographic image (Figure 5(b)). The trade-off between the highest OAM helical mode index and the resolution of the OAM-dependent holographic images sets up an upper limit of the maximum multiplexing channel number when a certain image resolution is specifically required [39]. In principle, enlarging the hologram size or reducing the reconstruction distance can help improve the image resolution, since the effective numerical aperture of the Fourier hologram can be increased.

Apart from the spatial-frequency sampling, the random phase of an OAM diffuser array also plays a key enabling role in the digitalization of a complex-amplitude hologram. Like the case of image processing with Fourier transform, the direct FT of an image usually gives rise to a complex-valued Fourier hologram with a large amplitude variation difficult to implement through a digital device (Figure 5(c)). The broad spatial-frequency content of a random phase function acts as a convolution kernel to diffuse the amplitude of a Fourier hologram, capable of flattening the large amplitude variation present in the Fourier hologram. To quantify the effect of the random phase on digitalization, the coefficient of amplitude variation as a function of OAM-multiplexing channel number was characterized in Figure 5(d). Without the OAM diffuser array, the coefficient of variation in the amplitude part of a Fourier hologram is nearly 300 % for a typical set of images and increases with the multiplexing channel number. This results in about 90 % area of the Fourier hologram having normalized amplitudes smaller than 0.05. This sets a fundamental barrier to digitalise a Fourier hologram due to the loss of phase information and low transmission efficiency (less than 2.5 %) in that area. After applying the OAM diffuser array, the coefficient of variation is reduced below 100 % and kept nearly constant by increasing the multiplexing channel number (Figure 5(d)). It was found that the random phase of an OAM diffuser array can also eliminate the coherence of image channels and further suppress multiplexing crosstalk in addition to the OAM orthogonality [149].

#### 3.2.2 OAM-selective and -multiplexing holograms

**Figure 6:**
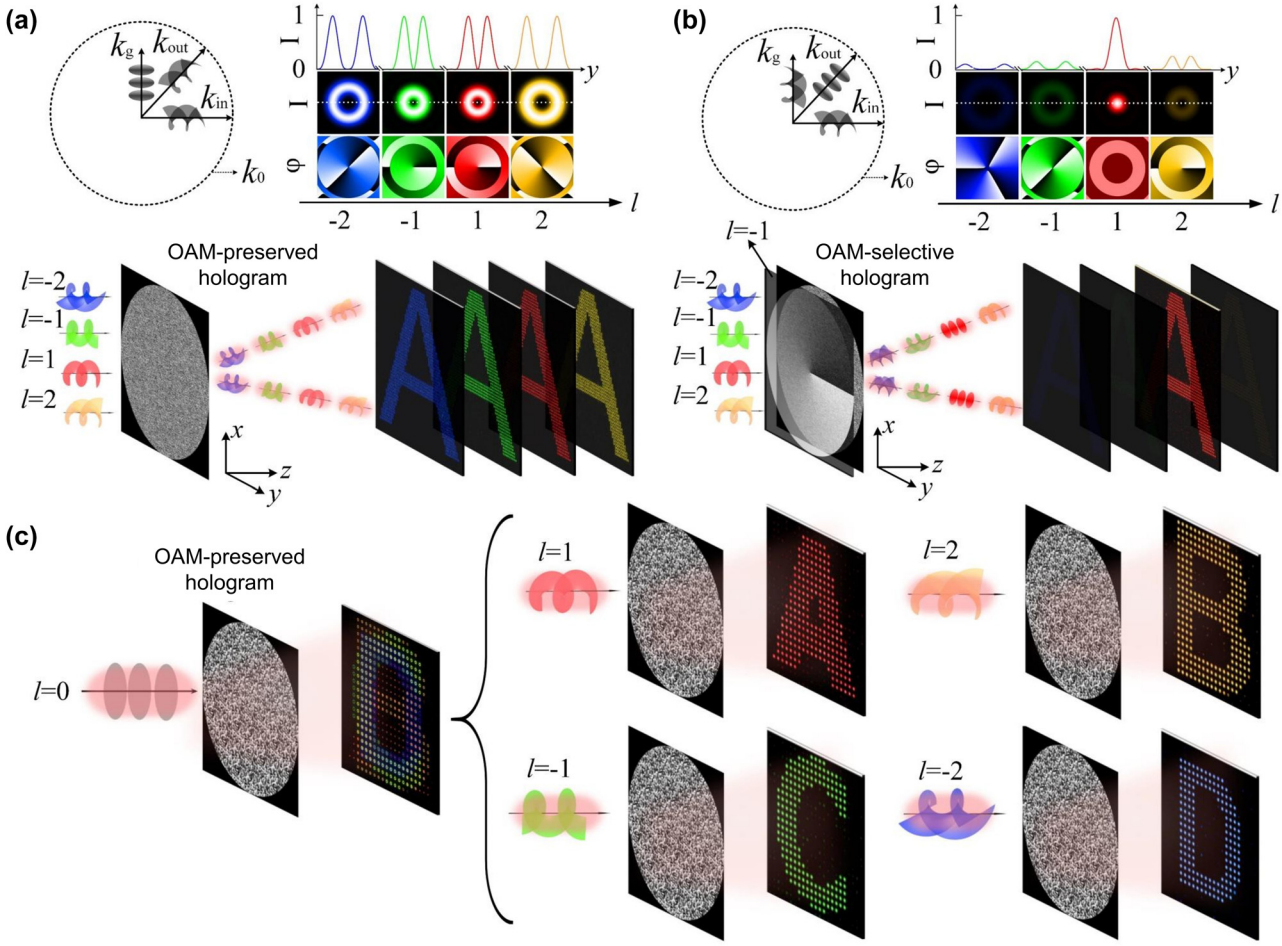
Schematic illustration of OAM-preserved, -selective, and -multiplexing holograms. Bottom (a, b): schematics of an OAM-preserved hologram (a) and an OAM-selective hologram (b) capable of transferring the OAM property from an OAM incident beam to a holographic image and of reconstructing given OAM channels, respectively. Top left (a, b): OAM transfer in conjunction with the linear spatial frequency shift (k-space). Top right (a, b): intensity (I) and phase (*φ*) distributions of single pixels selected from the reconstructed holographic images, where the holographic images highlight OAM pixels featuring a doughnut-shaped intensity distribution (a) and solid-spot pixels reconstructed from a given incident OAM beam (b), respectively. (c) Schematic of an OAM-multiplexing hologram capable of reconstructing OAM-dependent holographic images. Reproduced from Ref. [37] with permission from Nature Publishing, copyright 2019.

The principle of OAM holography is illustrated in Figure 6, where an OAM-preserved hologram with a discrete spatial-frequency distribution is designed to maintain the OAM property of incident OAM beams in each pixel of reconstructed holographic images (Figure 6(a)), laying the physical foundation for using OAM as an independent information carrier [37]. To achieve the OAM selectivity in holography, the phase function of a spiral phase plate with a topological charge of *l* can be further added onto the design of an OAM-conserving hologram, leading to an OAM-selective hologram (Figure 6(b)). In this context, holographic images appear only when an OAM beam with an inverse topological charge of −*l* is incident on an OAM-selective hologram. Such strong OAM selectivity originates from both the spatial and intensity distinctions between a fundamental spatial mode and high-order OAM modes in the image plane. Each pixel in the selectively reconstructed holographic images features a fundamental spatial mode dictated by a solid-spot intensity distribution with higher intensity value. To further improve the OAM selectivity, a fundamental mode filtering aperture array in the detector plane was added to rule out high-order OAM modes with doughnut-shaped intensity distributions. To demonstrate the OAM selectivity, four OAM-selective holograms (labelled as “1”, “2”, “3”, and “4”) were designed to confirm strong OAM selectivity (Figure 6(c)). It was shown that four different holographic images can be selectively reconstructed from the OAM-multiplexing holograms based on the incident OAM beams with topological charges of −2, −1, 1, and 2, respectively.

#### 3.2.3 Machine-learning and superresolution OAM holography

**Figure 7:**
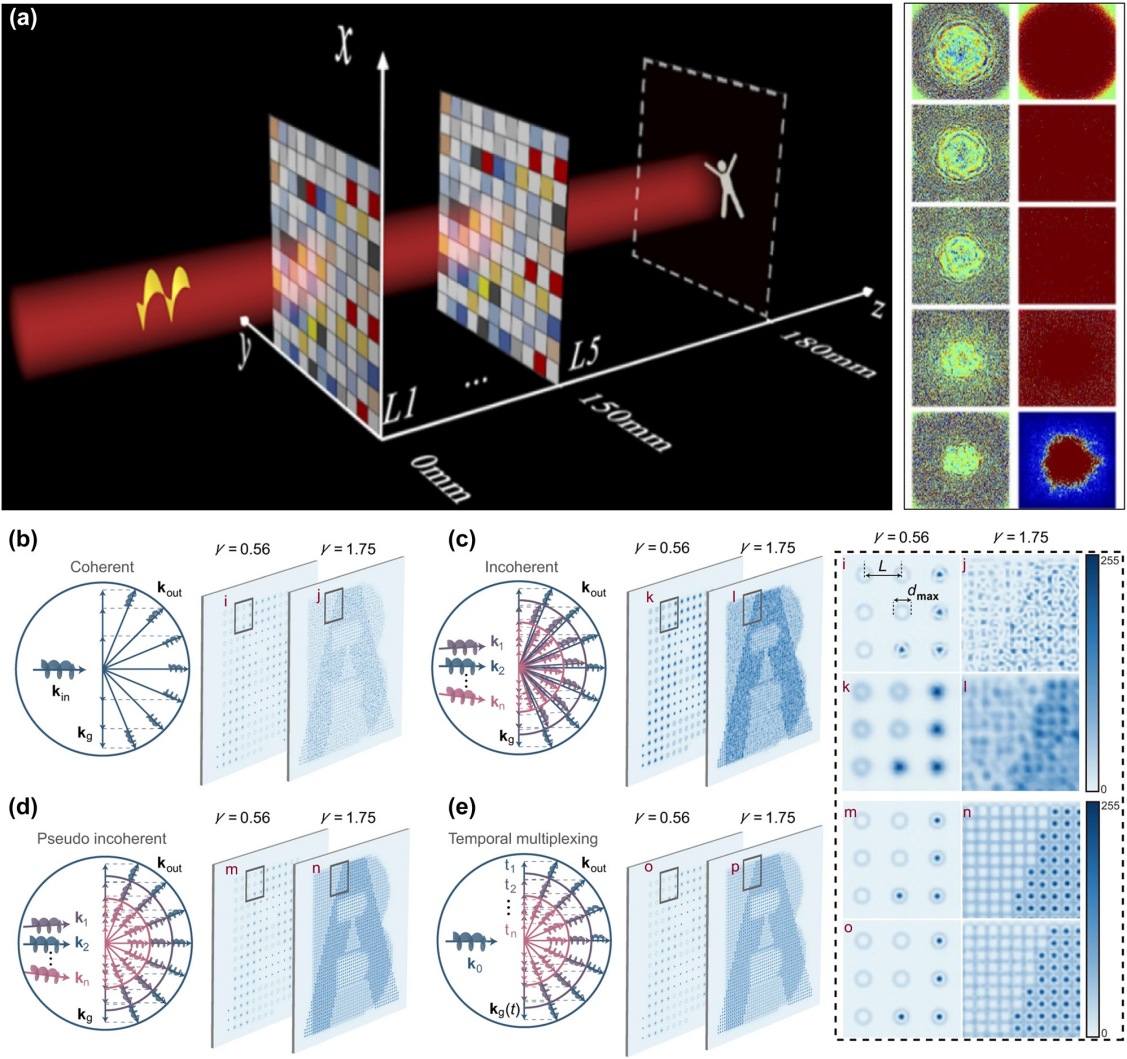
Principle of OAM holography based on an optical diffractive neural network (ODNN) and a pseudo incoherent approach. (a) Schematic illustration of OAM multiplexing holography based on a five-layer ODNN, wherein each incident OAM vortex mode can be converted to a kinetic action (KA)-based holographic image. Right column: calculated phase (left) and amplitude (right) distributions in the five layers ODNN model. Reproduced from Ref. [40] with permission from Optica, copyright 2022. (b–e) Schematic illustration of the OAM property transfer in the spatial frequency domain. Reconstructed images with two OAM channels are shown, where the enlarged areas (marked as *i* to *p*) are compared in the rightmost panel. (b) General coherent case. (c) General incoherent case. (d) Pseudo incoherent case. (e) Coherence suppression case with temporal multiplexing. (b–e) reproduced from Ref. [41] with permission from Nature Publishing, copyright 2023.

Even though OAM holography can be achieved from a spatial-frequency-sampled hologram, this approach inevitably leads to a sparse image with a trade-off between the hologram bandwidth (the number of OAM multiplexing channels) and the image resolution, as represented in Figure 5(b). This bottleneck in OAM multiplexing holography reveals that a single-layer diffractive structure cannot realize complex structured light field conversion. To address this key challenge in OAM holography, the idea of using multiple diffractive phase plates was recently proposed [40]. Multiplane light conversion (MPLC), a commercial technology that uses cascaded phase plates, has recently offered a viable approach to performing arbitrary transformations on spatial modes with low intrinsic loss and low crosstalk [150]. The phase profiles of the multiple diffractive phase plates can be numerically designed by the optical diffractive neural network (ODNN), exhibiting an excellent information processing ability. Recently, OAM multiplexing holography was implemented by ODNN. By employing the MPLC and *in-situ* optical forward/backward propagation principles, unitary transformation and linear phase modulation were implemented to simultaneously modulate multiple OAM channels, where OAM modes are encoded independently with holograms and converted during optical propagations. Huang et al. [40] showed that a 5-layer ODNN can realize simultaneous conversion and evolution of 10 OAM multiplexing image channels at 5 spatial depths, where the mean square error and structural similarity index measure were 0.03 and 86 %, respectively. In the proposed OAM multiplexing holography, the OAM modes are mainly modulated by the MPLC principle that is composed by multiple amplitude-phase modulated diffractive layers. Figure 7(a) shows a schematic diagram of a five-layer ODNN. Huang et al. utilized Laguerre–Gaussian beams to generate the OAM modes at a wavelength of 1550 nm. They trained a five-layer ODNN to construct the light field conversion between the OAM modes and kinetic action (KA) holograms with binary grayscale values. They selected 10 OAM modes (l ∈ [−5, +5], l ≠ 0) as inputs to encode 10 KA holograms. By feeding these 10 samples into the ODNN for training to minimize the loss function, the amplitude and phase parameters inside the diffractive layers were simultaneously calculated and updated owing to the backward propagation of gradients. After 8000 iterations, the ODNN can accurately convert all 10 OAM modes to the corresponding KA holograms at an output plane, and the loss function converges at approximately 0.06.

To reduce strong multiplexing crosstalk in OAM holography, the above introduced sampling criterion dictates that the image sampling distance should be no less than the diameter of largest addressable OAM mode, which severely hinders the increase in resolution and capacity. To mitigate this compelling challenge, an idea of multiplexing a range of OAM holograms in the time domain via a fast-switching digital mirror device was proposed [41], leading to a pseudo incoherent approach that largely relaxes the sampling constraint in OAM holography. Shi et al. [41] recently proposed a pseudo incoherent approach that is almost crosstalk-free, and demonstrated an analogous coherent solution by temporal multiplexing, which dramatically eliminates the crosstalk and largely relaxes the constraint upon sampling condition of OAM holography, exhibiting a remarkable resolution enhancement by several times, as well as a large scaling of OAM multiplexing capacity at fixed resolution. In principle, the ratio of the diameter of largest addressable OAM mode (dmax, denoting the OAM-multiplexing capacity) to the sampling distance (*L*, indicating the image resolution of OAM holography), *γ* = *d*_max_/*L*, is a core factor termed as the sampling condition. It is greatly desirable to enlarge the value of *γ* as much as possible for OAM holography, since it corresponds to higher resolution with a certain number of OAM channels, or equivalently higher capacity at a certain resolution. However, for the general coherent OAM holography as previously reported, OAM property cannot be preserved in densely sampled high-resolution images due to interference, thereby imposing a fundamental resolution limit for a given OAM channel number by demanding *γ* ≤ 1. Specifically, for the general coherent case (Figure 7(b)) that uses a coherent illumination source, the constituent spatial frequencies (*k*_g_ in the momentum space) of an OAM-conserving hologram add a linear spatial frequency shift to an incident OAM beam (*k*_in_). Considering a hologram that multiplexes two OAM channels as an example, a sparse sampling (*γ* = 0.56) can retain the OAM property of the incident beam (as denoted in the enlarged area *i*), however a denser sampling with *γ* = 1.75, which exceeds the sampling criterion limit, causes a complete loss of OAM property due to interference (see the enlarged area *j*). On the other hand, for the general incoherent case (Figure 7(c)) that uses an incoherent illumination source, the spatial frequencies of different incoherent wavefronts have deviated outgoing directions according to the diffractive grating formula, which brings serious undesired blurring to the reconstructed image (see the enlarged area of *l*).

Unlike the general incoherent case, a pseudo incoherent case (Figure 7(d)) assumes that the outgoing wave vectors leaving the hologram coincide perfectly for all incoherent incident vectors (*k*_1_ to *k*_*n*_), so that individual diffraction patterns can be superposed in an intensity manner (see the enlarged area of *n*) without introducing image blurring. However, this case is practically challenging to realize due to the violation of the dispersion of diffractive gratings; thus, a coherent analogy to that is alternatively carried out, by temporal multiplexing of a coherent beam, thereby suppressing the coherence. It is shown in Figure 7(e) that temporal multiplexing introduces time-varying phase delays to the incident vector, thereby creating multiple incoherent wavefronts within each modulating duration, which is akin to inherent multiple wavefronts in the pseudo incoherent case, and consequently enables high reconstruction quality at super-resolution condition as well (see the enlarged area of *p*). Therefore, temporal multiplexing, rather than a partially coherent light source, can be used to reduce the coherence of the reconstructed images, well-preventing damage in the structure of holographic images and OAM orthogonality. However, this temporal multiplexing method requires high-speed spatial light modulating devices such as digital micromirror device and ferro-electronic liquid crystal on silicon, thus restricting the available types of CGHs to amplitude binary holograms for digital micromirror device and phase binary holograms for ferro-electronic liquid crystal on silicon, respectively.

**Figure 8:**
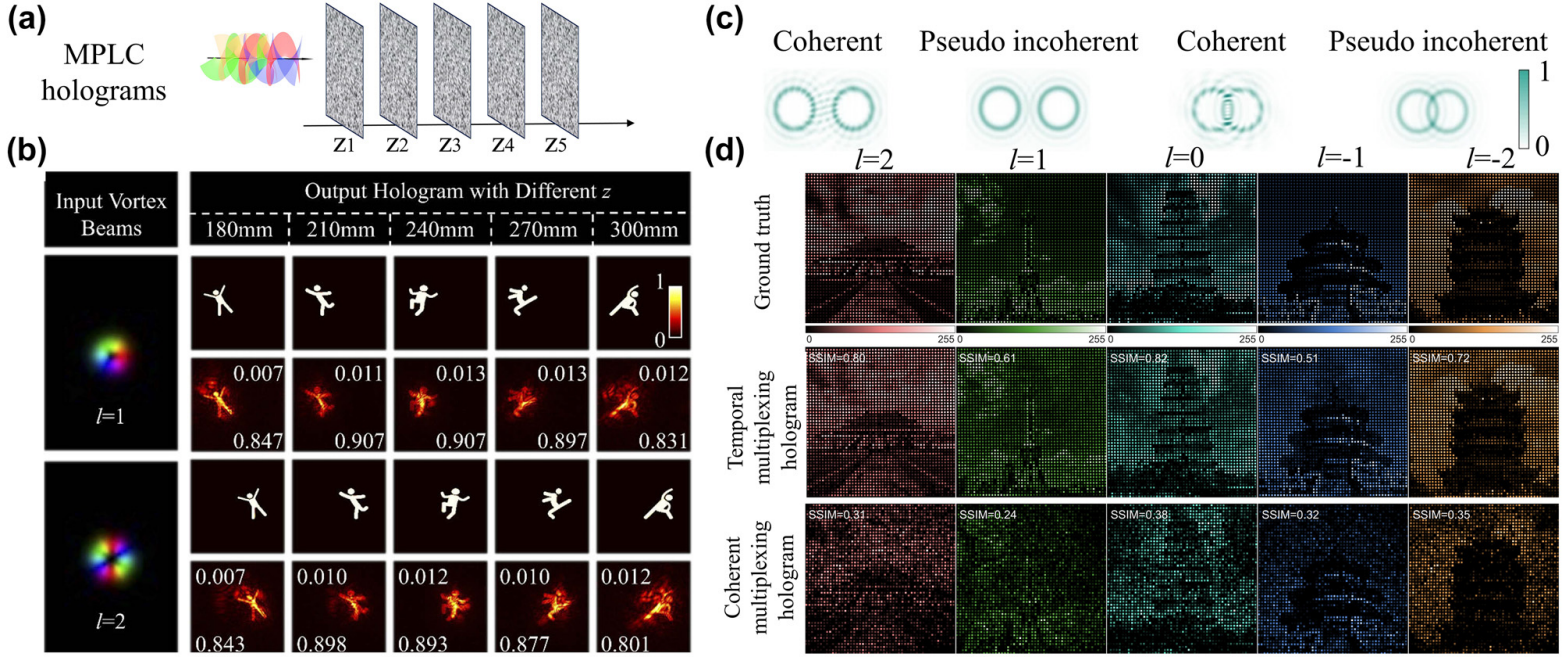
Holographic imaging results of the ODNN approach and of temporal multiplexing OAM holography. (a) and (b) Imaging reconstruction results of the ODNN approach based on five-layer MPLC holograms. (c) and (d) Pseudo incoherent approach based on temporal multiplexing of 50 binary OAM-multiplexed holograms on a digital micromirror device. MSE: mean square error. SSIM: structural similarity index measure. (b) Reproduced from Ref. [40] with permission from Optica, copyright 2022; (c) and (d) reproduced from Ref. [41] with permission from Nature Publishing, copyright 2023.

Figure 8(a) and (b) present the holographic imaging results of the ODNN approach based on five-layer MPLC holograms. The average mean square error (MSE) and structural similarity index measure (SSIM) for the two OAM modes of l = 1 and 2 at five spatial depths were 0.0107 and 87.01 %, respectively [40]. Even though this method allows the reconstruction of OAM-dependent holographic images with continuous image pixels not suffering from a sparse spatial sampling, there is huge room for improving the image quality and testing more complex gray scale image targets. Figure 8(c) and (d) present the experimental imaging results of densely sampled grayscale images for the temporal multiplexing OAM holography [41]. Figure 8(a) compares the results of two OAM pixels based on the coherent and pseudo incoherent approaches, with the sampling rate exceeding the size of diffraction-limited OAM pixels in the Fourier plane, as explained in Figure 4. For the coherent case, the two OAM pixels cause strong mode degradation due to interference. Five OAM-encoded holographic images with 256 gray levels of landmarks were faithfully reconstructed from 50 binary OAM-multiplexed holograms dynamically displayed on a digital micromirror device at 10 kHz (mid row in Figure 8(d). As a comparison, the experimental results obtained from a single coherent OAM-multiplexing hologram suffer from heavy noises that severely reduce the image quality.

### 3.3 Fabrication of metasurface holograms

**Figure 9:**
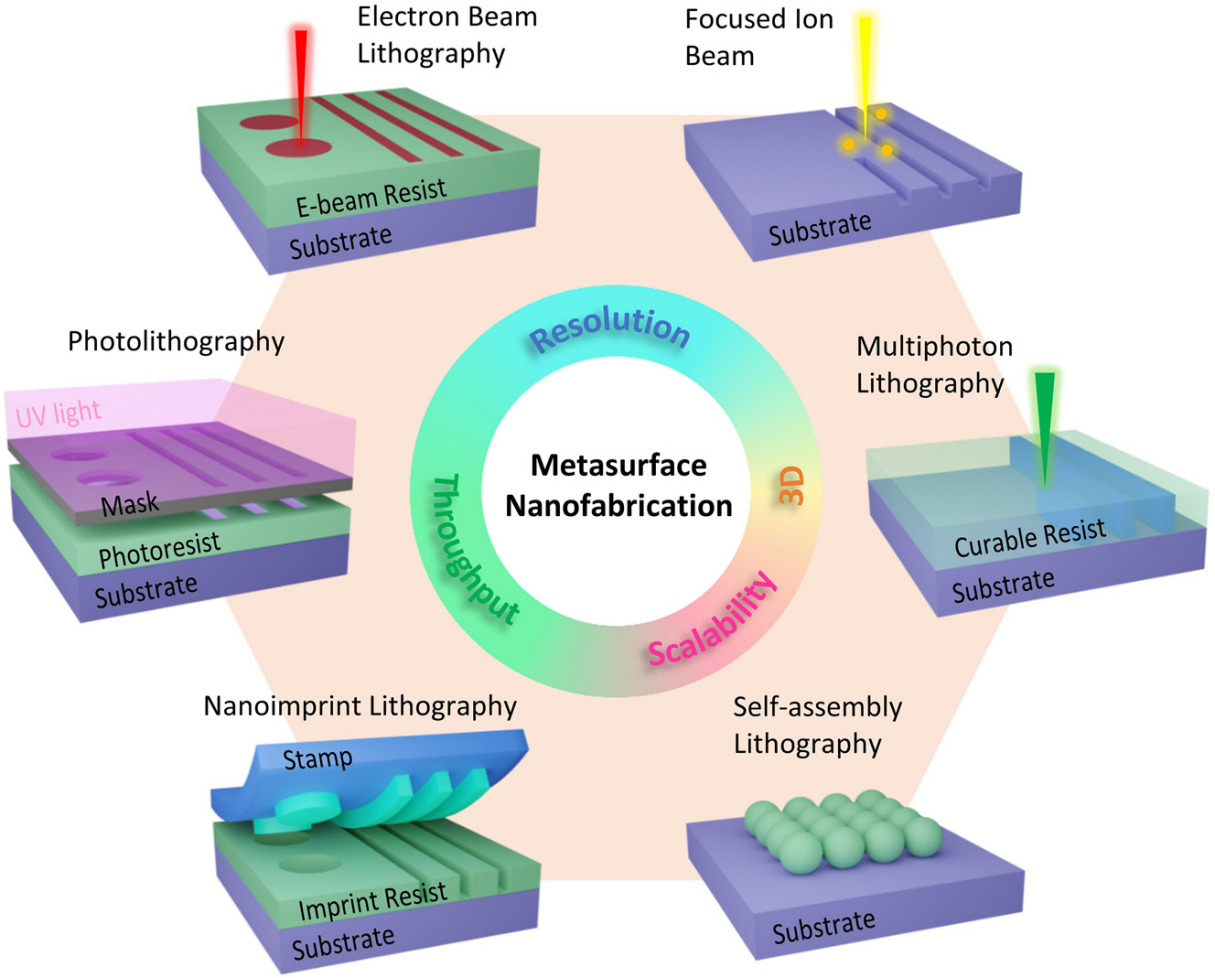
Nanofabrication techniques for metasurface holograms. Electron-beam lithography (EBL) and focused ion beam (FIB) are the mostly used maskless and high-resolution fabrication approaches. Photolithography and nanoimprint lithography are high-throughput and high-resolution fabrication approaches. The self-assembly method is a potentially affordable and scalable technique. The newly developed multiphoton lithography is another maskless technique that is superior in the direct writing of three-dimension (3D) structures.

Metasurface holograms comprise of subwavelength meta-atoms requiring advanced nanofabrication techniques to achieve high-quality fabrication performance. Figure 9 illustrates some of the frequently used fabrication methods, where six approaches are classified into four groups based on their fabrication features, namely resolution, throughput, scalability, and 3D capability. Since none of the single method can meet the various demands, all these approaches are used specifically depending on the tasks. For instance, to fabricate multilayer metasurfaces, one could do multilayer optical/e-beam lithography [151–153], or do aligned stacking with the transfer technology that been widely used in 2D materials [154]. The general mechanism behind these techniques is either lithography or direct writing, each has respective pros and cons.

#### 3.3.1 High-resolution lithographic techniques

Electron-beam lithography (EBL) system is similar to a scanning electron microscope, instead of scanning the full area for imaging purpose, electron beam is scanned in a controlled manner (via a blanker) so that computer-aided design patterns can be written to desired sample area with high accuracy. A more dedicated EBL working under high accelerating voltage (over 100 KV) enables the focal spot of a few nanometers leading to ultrahigh patterning resolution. EBL is likely to be one of the most frequently used nano fabrication methods in labs and research institutes due to its high resolution and flexibility of patterning. The EBL fabrication usually consists of three main steps, patterning, mask deposition and pattern transfer.

Patterning on the resist is the key step in EBL that dominants the resolution of fabrication. In this process, the chemical property of the polymer resist is modified by the secondary electrons and forming the designed structures after development. Specifically, first spin coats the e-beam polymer resists solution, poly (methyl methacrylate) for example, on a clean substrate. And remove the residual solvent by a short baking on a hotplate or an oven. Then the coated substrate is loaded to a high vacuum chamber for electron beam exposure. Predesigned patterns are drawn by the programed focused electron beam scanning across the resist. Note that the majority pristine high-energy electrons just pass through due to the high accelerating voltage and relative thin resist film. The secondary electrons and a few back-scattered electrons with a few electron-volts energy effectively interact with the polymer chemical bond and determine the resolution of the exposure [155]. The exposed resist area will either become soluble (positive resist) or become resistant (negative resist) to the developer. To achieve good defined patterns, not only these secondary electrons but also the near- and far-field back scattered electrons need to be considered into the effective total dosage. These can be corrected based on the beam and material parameters. After the development, we get our designed nanostructure pattern on the resist layer.

The pattern can be transferred to the target substrate by dry etching (chemically or physically). For good etching selectivity, a thin layer of metal (e.g., chromium, nickel, and tungsten) can be deposited as the hard mask. The unwanted metal and resist will be removed during the liftoff process. Specifically, metal deposited on the surface of the resist will be washed away while only metal that directly deposited in the pattern area (on the substrate) remains. The metal mask then goes through an etching process by reactive (or inert) gases plasma. Note that many parameters including ion species, plasma density and bias voltage and so on need to be optimized to get desired etch rate and geometry profile. After etching, the pattern is finally transferred to the substrate. The metal mask is then chemically removed by corresponding remover solution. For metasurface holograms in the visible range, one can also consider using the SiO_2_, or negative resist hydrogen silsesquioxane (HSQ) as the mask, as the mask removal is less important in this scenario [156].

Like focused electron beam, focused ion beam (FIB) also can be used to do nanoscale precision fabrication. Unlike EBL, FIB is capable of material “deposition” and “milling”. For deposition, the gas precursor molecules are adsorbed on the surface, and then are decomposed by the ion-beam. Meanwhile, the high energy and large cross section property of the ions enable milling the substrate or target materials directly. FIB fabrication is based on the principle of ion-solid interactions, which causes sputtering, milling or deposition processes depending on the beam current and gas chemistry. There are a large range of ion species available for different purposes. Light ions such as helium, lithium, nitrogen can be used to achieve high lateral resolution and deep penetration while heavy ions like argon, gallium, and xenon are suitable for milling on hard materials.

Sub-10 nm resolution is easy to access for both EBL and FIB [157]. The high resolution mostly comes from the ultra-small beam spot size. Electron beams can be focused to sub-one nm, intrinsically smaller than the point spread function of extreme ultraviolet (UV) light source (13.5 nm wavelength) which is the most advanced and important light source for photolithography. Considering the meta-atom size, a beam spot of a few to 10 nm is sufficient for the visible and longer spectra range. The cost of high resolution and precision is low scalability and throughput, as the pattern must be written individually.

#### 3.3.2 High-throughput lithographic techniques

It is the high throughput instead of high resolution that greatly impacts the manufacturing of metasurface holograms. Products like semiconductor chips and book presses are the well-known typical examples that benefit from mass production. Technologies behind these are photolithography and imprint lithography. As is known, in photolithography, wide-field illumination is applied to create the patterns with the help of predesigned photomasks. Therefore, the total resolution relies highly on light source and masks (molds for the imprinting). High-energy photons trigger the chemical reaction in photon sensitive resists (i.e., photoresist) just like the low-energy secondary electrons that trigger the chemical reaction of e-beam resists in EBL process. Short wavelength light (*λ*) in the UV range is desired source for photolithography due to lower diffraction limit, about *λ*/2*n*, where *n* is the real part refractive index of the medium. To increase the resolution, we either increase the *n* or decrease the *λ*. However, bright and short-wavelength light sources are expensive, plus the corresponding optical components that work in the deep and extreme UV range further increase the expense. For the photomasks, it is usually an opaque plate with transparent areas that allows light to pass through, it can be fabricated by EBL or FIB. Apart from the use of mask and the large-area exposure, photolithography follows the same work procedure as EBL, namely the development, lift-off, etch, etc.

Imprint lithography may be the oldest and simplest form of expression of human that our ancestors used to paint objects like hands, feet, and tools on soft surfaces. It evolved a lot as civilizations. For example, the use of metal stamps and seals enabled the creation of more durable and precise imprints on various media such as wax, paper, leather, or wood. The invention of movable type and printing press revolutionized the production and dissemination of written texts and images. Nowadays, it is even more useful and powerful. The developed nanoimprint lithography (NIL) has emerged as a promising candidate to fabricate metasurfaces. Specifically, it is potentially of high resolution, high-throughputs, scalable and low cost at the same time. NIL can be classified into two main types: thermal imprint and UV imprint. Thermal imprint uses heat and pressure to deform a thermoplastic polymer film, while UV imprint uses a UV-curable resin and UV light exposure to harden the pattern [158]. Since both methods need the master molds to transfer the structures or pattern. Sub-10 nm feature sizes and high throughput can be achieved only if molds are prefabricated properly. Rho’s group and their collaborators remarkably demonstrate wafer-scale metasurfaces fabricated via nanoimprint lithography with a critical dimension of ∼40 nm [159]. It is potentially a desired next generation affordable high-throughput technique. However, at this stage, it also faces some challenges, such as mold fabrication and alignment, defect control and demolding.

#### 3.3.3 Highly scalable fabrication techniques

Self-assembly nanofabrication is a bottom-up approach that uses chemical or physical forces at the nanoscale to assemble basic units into larger structures or patterns with desired properties and functions. It is inspired by biological systems that create complex structures through self-organization of molecular components. One of the methods for creating nanostructures on various substrates is colloidal lithography, which uses self-assembled spherical monolayers as masks for etching and deposition [160]. Polymer spheres such as polystyrene, which can form large-scale and nearly defect-free monolayer hexagonal close-packed arrangements on the water−air interface or by using a Langmuir−Blodgett trough [161]. Colloidal lithography is a simple and efficient technique that does not require expensive equipment or clean-room facilities. The spacing and density of the particles in a monolayer can be tuned by varying the length of polymer chains on the nano-particle surface and the particle concentration, respectively. The assembled nanoparticles can transfer the pattern to the substrate by direct etch or further combine vacuum deposition to transfer the pattern to a hard mask and followed by dry etch [161].

Some examples of self-assembly nanofabrication methods are inverse micelles [29], quantum dots [162], carbon nanotubes [163], and DNA-assisted assembly [163, 164]. These methods can produce nanoparticles, nanowires, nanotubes, and nanodevices for various applications in electronics, optics, magnetism, biotechnology, and medicine. Self-assembly nanofabrication offers several apparent advantages over top–down techniques, such as low cost, high throughput, and precise control over size, shape, and composition of the nanostructures.

#### 3.3.4 3D laser nanoprinting technique

The above techniques are broadly and effectively used to determine the lateral features of nanostructures. To shape structures in 3D in these technologies, processes like layer-by-layer approach are needed. Given the fabrication process and nanoscale dimensions, the alignment precision between layers is challenging and the complexity is increased inevitably. 3D laser printing in transparent materials has becoming popular, especially multiphoton lithography (MPL) that allows the fabrication of 100-nm features. MPL uses ultrafast (femtosecond) laser pulses to create small three-dimensional structures. The principle of MPL is based on the nonlinear absorption of photons by a photosensitive material, such as a photoresist (usually polymers). It is originally predicted by Maria-Gőppert-Mayer in 1931 [165]. The photon energy of the light source is lower than the materials bandgap (e.g., an infrared laser), so it is transparent to the pristine light source, no spontaneous absorption. However, when the laser is confined into a small volume (i.e., voxel, the 3D analogy to 2D pixel), the photon density increase greatly, two or more photons can be simultaneously absorbed by the material, initiating a chemical reaction that changes the resist properties. Since the laser intensity maxima is located solely at the focal volume, direct writing in the material at arbitrary depth is achieved. By scanning the laser focus in a controlled manner, computer designed complex 3D patterns can be written in the material with sub-diffraction-limited resolution. Post processes like annealing and etching may further reduce the structure size hence increase the resolution [166, 167]. Another straightforward way of higher resolution is to increase the photons in the nonlinear process, like three-photon polymerization [168]. At present, two-photon polymerization (2 PP) is the main MPL, where near infrared (780 nm–800 nm) femtosecond lasers are used. The simplest 2 PP setup consists of a laser source, a focusing objective, a translational stage (or scanning mirror), a laser power control system, and a shutter. It is very similar to a conventional confocal system.

MPL has several advantages over conventional lithography methods, such as noncontact and maskless fabrication, high spatial resolution, and low environmental impact MPL has facilitated fields like nanophotonics [30, 169], microfluidics [170] and bioengineering [171, 172]. In addition, it allows direct printing of refractive and diffractive optical elements, and recently ultrathin metasurfaces on optical fibres and camera pixels [173–180] However, limitations exist as well. One of the main limitations is the trade-off between resolution and fabrication speed. In addition, limitation in the choice of suitable materials and photo-initiators that can support multi-photon absorption and polymerization, while being compatible with the desired properties and functionalities of the 3D structures. A third limitation of MPL is the difficulty of integrating other components or devices into the 3D structures. Therefore, developing new materials, photoinitiators and integration techniques that can overcome these limitations are important research directions for MPL.

Metasurface is a versatile platform that can be applied to any solid material. Up to date, numerous materials have been investigated, including both metals and dielectrics. High-quality single crystals may be required in some circumstances, for example, to achieve less loss. In most times, amorphous materials are expected, due to the more controllable and flexible in both thickness and dimensions. Table 2 lists some of the frequently used materials for metasurface devices. We note the size of the metasurface varies dramatically, as it is mainly restricted to the material growth methods and the patterning technologies as we introduced above. For example, wafer-scale single crystals could be a challenge for most materials, but their amorphous phases are usually not an issue. Additionally, when time cost is considered, up to a few millimeter or centimeter scale is reasonable for EBL and FIB, while photolithography and nanoimprint can easily reach wafer scale [207].

**Table 2:** Common host materials for metasurfaces.

Spectral range	Materials	Ref.
Ultraviolet	HfO_2_, Nb_2_O_5_, AlN, Si_3_N_4_, ZrO_2_, ZnO, Si, etc.	[181–186]
Visible to NIR	Ag, Au, Al, Cu, Cr, graphene, Si and a-Si:H, SiN, SiC. HSQ/SiO_2_, TiO_2_, VO_2_, hBN, diamond, perovskite, TMDCs, GaN, GaP, PMMA and other TPP resins, etc.	[174, 187–206]
Mid-infrared	Si, Al, Pt, Cu, PbTe, Ge, etc.	[207–211]

## 4 Perspectives and summary

Holography has been identified as one of key platforms for realizing 3D displays, optical encryption, microscopy, data storage, LiDAR, and artificial intelligence. We have provided a comprehensive overview of optical holography, moving from volume holography based on optically thick holograms to digital holography using ultrathin metasurface holograms in nanophotonics. We have reviewed the use of volume holograms for holographic multiplexing through linear momentum selectivity and other approaches. Information can be encoded using various properties of light. As such, metasurface multiplexing holography harnessing the degrees of freedom of light, including polarization, wavelength and incident angle have been reviewed in detail. However, a single hologram bandwidth has remained too low for practical use. To overcome this limitation, information can now be stored in the OAM of light, as this degree of freedom has an unbounded set of orthogonal helical modes that could function as information channels. We reviewed the principle of OAM holography and showcased its first realization from phase-only metasurfaces, which, however, are marred by channel crosstalk. To reduce multiplexing crosstalk, we further reviewed the design and fabrication of a complex amplitude metasurface capable of complete and independent amplitude and phase manipulation. Alternatively, machine learning algorithms and a pseudo incoherent approach have recently been proposed to deal with the multiplexing crosstalk in OAM holography. In the end, we reviewed current trends of using different nanotechnology methods for the fabrication of metasurface holograms. We highlight the use of maskless and high-resolution EBL and FIB fabrication approaches. For increase the fabrication throughput and scalability, we reviewed the use of highly affordable self-assembly method and nanoimprint lithography with high resolution. In addition, we highlight the use of 3D laser nanoprinting based on multiphoton lithography for direct writing of 3D structures on different substrates including optical fibres. We believe that nanophotonics science and nanofabrication technology will continue to make significant impact on future development of ultrathin, ultracompact, ultrahigh-bandwidth, and affordable holograms.

Future directions and challenges in optical holography are driven by the applications that it can uniquely cover such as AR see-through devices and 3D displays, wavefront engineering and optical encryption both in analog and digital holography, and optical data storage. In the case of 3D image, holographic optical elements are good candidates to solve the vergence-accommodation conflict while providing large eyeboxes and proper eye relief. In holographic data storage, nowadays most research is focused on the collinear geometry, which might be a more robust approach for commercial devices, with Bragg-based multiplexing methods applied combining analog and digital holography. And in wavefront engineering and optical encryption the possibilities are endless, since holographic methods, with photosensitive materials, spatial light modulators, and recent trend of using flat meta-optics, enable to locally modify the amplitude, phase and polarization of the light beam, and to simultaneously multiplex various functions into the same light beam, thus being a tool serving future advancement of modern photonics and optics-related applications. Therefore, in all these areas we expect to see many new conceptual developments and fabrication techniques with a strong impact in the holography field in coming years.
